# Commissioning and implementation of an implantable dosimeter for radiation therapy

**DOI:** 10.1120/jacmp.v14i2.3989

**Published:** 2013-03-04

**Authors:** Ivan Buzurovic, Timothy N. Showalter, Matthew T. Studenski, Robert B. Den, Adam P. Dicker, Junsheng Cao, Ying Xiao, Yan Yu, Amy Harrison

**Affiliations:** ^1^ Department of Radiation Oncology Jefferson Medical College Kimmel Cancer Center, Thomas Jefferson University Philadelphia PA USA

**Keywords:** implantable dosimeter, commissioning, MOSFET, image‐guided radiotherapy

## Abstract

In this article we describe commissioning and implementation procedures for the Dose Verification System (DVS) with permanently implanted *in vivo* wireless, telemetric radiation dosimeters for absolute dose measurements. The dosimeter uses a semiconductor device called a metal–oxide semiconductor field‐effect transistor (MOSFET) to measure radiation dose. A MOSFET is a transistor that is generally used for amplifying or switching electronic signals. The implantable dosimeter was implemented with the goal of verifying the dose delivered to radiation therapy patients. For the purpose of acceptance testing, commissioning, and clinical implementation and to evaluate characteristics of the dosimeter, the following tests were performed: 1) temperature dependence, 2) reproducibility, 3) field size dependence, 4) postirradiation signal drift, 5) dependence on average dose rate, 6) linearity test, 7) angular dependence (different gantry angle position), 8) angular dependence (different DVS angle position), 9) dose rate dependence, 10) irradiation depth dependence, 11) effect of cone‐beam exposure to the dosimeter, and 12) multiple reading effect. The dosimeter is not currently calibrated for use in the kV range; nonetheless, the effect of the cone‐beam procedure on the MOSFET dosimeter was investigated. Phantom studies were performed in both air and water using an Elekta Synergy S Beam‐Modulator linear accelerator. Commissioning and clinical implementation for prostate cancer patients receiving external‐beam radiation therapy were performed in compliance with the general recommendations given for *in vivo* dosimetry devices. The reproducibility test in water at human body temperature (37°C) showed a 1.4% absolute difference, with a standard deviation of 5.72 cGy (i.e., SD=2.9%). The constancy test shows that the average readings at room temperature were 3% lower compared to the readings at human body temperature, with a SD=2%. Measurements were not dependent upon field size. Due to postirradiation signal drift, the following corrections are suggested: −2.8%, −2%, 0.5%, and 2.5% for the readings taken after 0.5, 1, 5, or 10 min, respectively. Different gantry angles did not influence the readings. The maximum error was less than 1% with a maximum SD=3.61cGy (1.8%) for the gantry angle of 45°. However, readings are dependent on the dosimeter orientation. The average dose reading was 7.89 cGy (SD=1.46cGy) when CBCT imaging was used for the pelvis protocol, and when postirradiation measurement was taken at 2.5 min (expected 2–3 cGy). The clinical implementation of the implantable MOSFET dosimeters for prostate cancer radiation therapy is described. Measurements performed for commissioning show that the dosimeter, if used within specifications, provides sufficient accuracy for its intended use in clinical procedures. The postradiation signal drift, temperature dependence, variation of reproducibility, and rotational isotropy could be encountered if the dosimeter is used outside the manufacturer's specifications. The dosimeter can be used as a tool for quantifying dose at depth, as well as to evaluate adherence between planned doses and the delivered doses. Currently, the system is clinically implemented with ±7% tolerance.

PACS numbers: 87.53.‐j; 87.55.‐x

## I. INTRODUCTION

There was significant interest recently in investigating direct tumor *in vivo* dosimetry and dose verification using novel technologies.^(^ ^1^ ^–^ ^11^ ^)^ Initial research effort focused on comparing the doses derived from the signal of detectors placed on the skin to the theoretical values as calculated by the treatment planning system (TPS) for radiation therapy (RT). External dosimetry (often called *in vivo* dosimetry) is a quality control check on the radiation treatment plan and the equipment used to administer radiation. It is designed to catch any gross calculation or setup errors.

A more challenging approach for *in vivo* dosimetry is measuring the dose inside the target volume to verify the correct delivery of RT. The deviation between calculated and measured doses inside the target volume may offer an additional analysis of RT techniques and delivery. This may help researchers and clinicians better understand the side effects of RT and may lead to changes in treatment planning or delivery.

The Dose Verification System (DVS) (Sicel Technologies, Inc., Cary, NC) uses an implantable dosimeter and an external reading system to measure the absorbed dose near a tumor within a patient. The purpose of this study was to commission the DVS permanent implantable *in vivo* dosimeter system, and to acquire and evaluate sufficient data to ensure standards of quality in a clinical setting. The implantable dosimeter measures *in vivo* dose from photon external beam therapy. The dosimeter uses a semiconductor metal–oxide semiconductor field‐effect transistor (MOSFET) to measure radiation dose. Generally speaking, a MOSFET is a transistor used for amplifying or switching electronic signals. This implantable dosimeter can be used, together with current planning and treatment delivery techniques, to help improve the accuracy of dose delivered to radiation therapy patients. The dosimeter‐based approach may be valuable to validate doses delivered in patients, and the implanted dosimeters can also be used as fiducials markers for image‐guided (IG) RT.

The dosimeter uses a system similar to commercially available radio frequency identification (RFID) tags to transmit data. RFID is a technology that communicates through radio waves to exchange data between a reader and an electronic tag attached to an object for the purpose of identification and tracking. The dosimeter is powered by radio frequency telemetry, eliminating the need for a power source inside the dosimeter. The data can be accessed telemetrically for each treatment day during the course of therapy. Unlike RFID tags that only transmit an identification number, the dosimeter transmits a signal that is converted to dose by the reader to report the absorbed dose to tissue at the sensor location.^(^ ^12^ ^)^

The DVS is a permanent, implantable wireless, telemetric radiation sensor for absolute dose measurements. The active area of the DVS dosimeter is 1.3 mm from the apex of the biocompatible glass capsule, a copper antenna is at the base, and the capsule is 2.0 cm in total length. The DVS dosimeters have preset calibrations from the manufacturer with the intention to be used at 37°C.

The DVS system is specifically indicated for breast and prostate cancer to measure patient dose in photon beam therapy, and to serve as an adjunct to treatment planning to permit the measurement of the *in vivo* radiation dose received at the site of the implant (e.g., tumor periphery, tumor bed and/or surrounding normal tissues) for validation of the prescribed dose.

**Figure 1:**
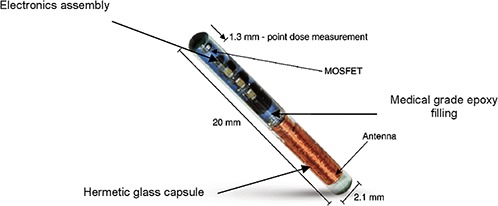
Implantable dosimeter: design and parts. Image supplied by Sicel Technologies, Inc. (Cary, NC).

The implantable dosimeter DVS (Fig. 1) consists of several parts: a) hybrid electronic assembly containing a radiation‐sensing field‐effect transistor (RADFET), an application‐specific integrated circuit (ASIC), and a support circuitry mounted to a ceramic circuit board; b) MOSFET; c) internal filling of medical grade epoxy; d) bidirectional antenna coil which provides the dosimeter with power and communications channels (the antenna is visible in the kV images); e) laser sealed, hermetic, biocompatible glass capsule (2.12 mm diameter, 20 mm length).

The following outlines the basic principles of operation. Radiation causes charge to be trapped in the layer of oxide in the MOSFET. The trapped charge changes the threshold voltage. The shift in the threshold voltage is proportional to the radiation dose deposited in the oxide layer. A correlation is made which establishes the relationship between the amount of trapped charge (due to radiation) and the voltage shift in the MOSFET. The calibration factor for the detector determines the relationship between the voltage shift and the amount of radiation.

DVS dosimeters are delivered by the manufacturer in pairs. The sensitive volume is 0.3mm×0.05mm. The dosimeters are factory calibrated using cobalt‐60. Recommended implantation depth is 3–12 cm maximum from any one skin surface. It was recommended that the distance between two dosimeters should be at least 1 cm. The dosimeters are manufactured to be used in the energy range of 6–18 MV photons and up to 8,000 cGy. To assure accurate readings, daily dose per fraction should be in the range of 150–250 cGy. The recommended time to read the dosimeter is up to 10 minutes following radiation therapy, with the optimal time being 2 to 3 minutes after radiation therapy. The dosimeter is optimized for use *in vivo* at temperatures ranging from 34°C to 40°C. Dosimeter readings at room temperature are 3%–5% lower compared to the ones for the calibrated temperature range. Approved shelf life, based on validation of sterility for clinical implantation in humans, is 24 months. Dosimeters can be stored at temperatures from $-18^{0}C$ to 55°C, and should be kept in a dark place where humidity is in the range of 10%–95%. Finally, the vendors' specifications for dose accuracy are <5.5% (2 σ) up to 2,000 cGy; <6.5% (2 σ) up to 7,004 cGy. The accuracy decreases slightly above 7,400 cGy. There is some rotational isotropy for the dosimeters, if a single field is used, up to ±1.5%.

Many studies on implantable dosimeter have been published recently.^(^ ^13^ ^–^ ^22^ ^)^ The phantom testing performed to study the radiation characteristics and accuracy of the response of this dosimeter^(^ ^13^ ^)^ described the functionality, radiation characteristics, and possible clinical implementation of an implantable MOSFET radiation detector (dosimeter). A rigorous *in vitro* calibration methodology was reported.^(^ ^14^ ^)^ In one pilot study,^(^ ^15^ ^)^ the authors discuss safety, efficacy, and utility of the device for ten patients with unresectable cancers of a variety of anatomic sites. The accuracy of the dosimeter was analyzed in the article by Beddar et al.^(^ ^17^ ^)^ Another study^(^ ^18^ ^)^ analyzes the results of 29 prostate patients from three clinical trial sites: prostate, nonprostate tissue, and in a region of anticipated dose falloff. Several research groups investigated the possibility of using a MOSFET dosimeter as a fiducial marker for IGRT^(^ ^20^ ^,^ ^21^ ^)^ or an application in robotic radiation therapy.^(^ ^22^ ^)^

In the current study, we add to the literature base for DVS by presenting the results of rigorous measurements of DVS characteristics in different clinical situations designed to simulate clinical care, such as dosimeter migration, dosimeter angulations, decay of signal over time, and measurements at body temperature. We also describe the basics of implantation program and policies adopted to use DVS for prostate cancer RT at our institution.

## II. MATERIALS AND METHODS

Radiation within the human therapeutic dose range causes a shift in the threshold voltage of the MOSFET. By measuring the threshold voltage before and after radiation therapy, the daily dose delivered at the MOSFET can be calculated. The cumulative dose can be calculated by tabulating the radiation dose measured at each fraction. To measure the absorbed dose, both predose and postdose readings must be performed. The predose threshold voltage reading is then subtracted from the postdose threshold voltage reading to calculate the daily dose for each treatment session. The daily dose values are then stored in the dosimetry database and are added together to calculate a cumulative dose during the RT course. This section describes the series of measurements performed to evaluate the DVS system under conditions designed to mirror clinical care.

### A. Experimental setup

Phantom studies were performed in both air and water using the Elekta Synergy S Beam Modulator linear accelerator (Elekta Ltd., Crawley, U.K.). As recommended by the vendor, the DVS dosimeters were placed at depths greater than $d_{\max}$, typically clinical depths of 5 cm and 10 cm for all tests, except for the irradiation depth dependence test. The field size was 10.4×10.4 cm (reference field size for the Elekta Synergy S linear accelerator), except for the field size dependence test. The quantity of measurements was established to generate statistically significant data for analysis.

#### A.1 Linear accelerator

**Figure 2:**
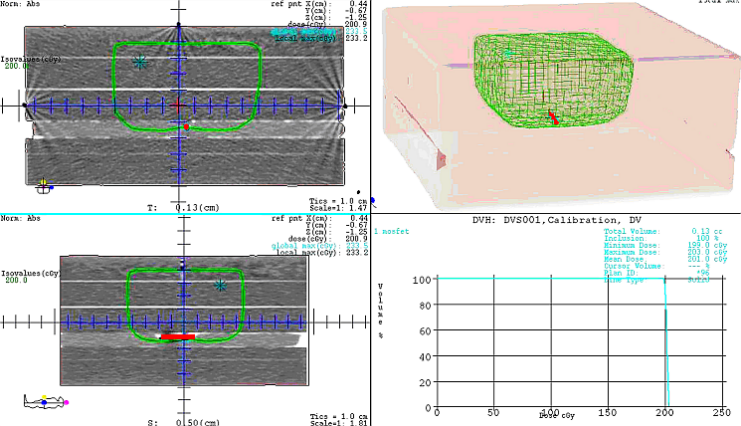
Dose calculation to a measuring point using solid water phantom; red marks the DVS.

To perform the set of the experiments we have used the Elekta Synergy‐S linear accelerator with cone‐beam imaging system. Two photon energies (6 MV and 10 MV) were used for the commissioning experiments. To evaluate the influence of the radiation in kV range to the implantable dosimeter, we have used the 3D X‐ray volume imaging (XVI) system incorporated into the linear accelerator. For all tests, the linear accelerator is adjusted to deliver 200 cGy to the point of measurement which is at the distance of 1.3 mm from the top of the dosimeter, as in Fig. 2. The figure represents a treatment beam where 200 cGy isodose line was normalized to the dosimeter measuring point.

#### A.2 Experimental phantoms

Temperature dependence, irradiation depth dependence, and absolute dose measurements were performed in water using 1D Scanner, Sun Nuclear's water phantom (Sun Nuclear Corporation, Melbourne, FL). The device has provision for automatic water level detections which removes the setup subjectivity. The phantom is pedant driven with 30 cm scanning depth. Since DVS dosimeters are photosensitive, when a transparent water phantom was used, the room lights were dimmed during irradiation and the readout process.

For other measurements, we used Gammex solid water (Gammex Inc., Middleton, WI) phantoms, which have characteristics to scatter and attenuate diagnostic and radiotherapy range X‐rays similar to water.

**Figure 3:**
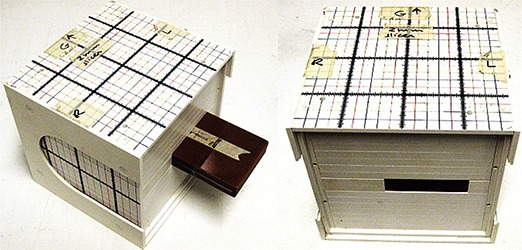
Experimental setup: solid water phantom and box with the dosimeter inside.

In the first case, the DVS was mounted into the holder and was immersed inside the water at an appropriate depth. For the second case, the DVS was placed inside the solid water box which is placed into the solid water phantom, as in Fig. 3, with a provision to change the depth of irradiation.

### B. Measurements

The daily dose measurement process contains several steps: taking a preirradiation reading, measuring preirradiation MOSFET threshold voltages, administering radiation, taking postirradiation reading, and measuring postirradiation MOSFET threshold voltages. The MOSFET voltage information is converted to a dose using dosimeter‐specific calibration information stored in the DVS database. Daily dose is reported in cGy.

For the purpose of acceptance testing, commissioning, and clinical implementation and to evaluate characteristics of the dosimeter, the following tests were performed: 1) temperature dependence, 2) reproducibility, 3) field size dependence, 4) postirradiation signal drift, 5) dependence on average dose rate, 6) linearity test, 7) angular dependence (different gantry angle position), 8) angular dependence (different DVS angle position), 9) dose rate dependence, 10) irradiation depth dependence, 11) effect of cone‐beam exposure to the dosimeter, and 12) a multiple reading effect. The dosimeter is not currently calibrated for use in the kV range. Nonetheless, the effect of the cone‐beam procedure to the dosimeter was investigated. Acceptance and commissioning were performed in compliance with the general recommendations.^(^ ^23^ ^)^ Due to the special characteristics of the dosimeter, additional tests were included in the commissioning procedure. For all tests we used four different dosimeters. Some of the tests, such as reproducibility, linearity, and water measurements, were performed multiple times to validate the results over a set of sensors to give the results sufficient statistical significance. To maintain sufficient accuracy level, the dosimeters used in this study were not previously irradiated and during the experiments the cumulative dose did not exceed the specification, except in the case where that was the intention of the experiment (e.g., reproducibility test). More specifically, the previous cumulative dose for the temperature dependence test, reproducibility test, linearity test, and effect of cone‐beam exposure test were zero. Excluding the reproducibility test, the maximum received dose received by the dosimeters was 7000 cGy at the end of the experiments, which was less than the 8000 cGy dose recommended by the manufacturer.

#### B.1 Temperature dependence

Dosimeters have been calibrated to give sufficient accuracy in the temperature range from 34°C to 40°C. We designed temperature tests to assess the dosimeter behavior at room temperature using solid water phantom, for the average body temperature of 37°C, and another temperature outside the calibration range at 41°C. The tests at 37° and 41°C were performed in the water phantom. Since the dosimeters are designed to allow one measurement per day, we used a water heater Haake D8‐L (Rheology Solutions Ltd, Victoria, Australia) with an immersion circulator to keep the temperature constant during the measurements. TPS (XiO 4.62, CMS Inc., St. Louis, MO) was used to calculate the monitor units to irradiate the dosimeter with 200 cGy by defining the dose point at the measurement point of the DVS. This method was also used by Scarantino et al.^(^ ^15^ ^)^ where the measured values were compared to values predicted by the TPS.

#### B.2 Reproducibility

A reproducibility test is usually performed with ten readings.^(^ ^23^ ^)^ For this study, we irradiated the dosimeter 50 times (200 cGy per fraction) in order to test the reproducibility over the whole course of prostate treatment (e.g., 44 fractions), which exceeds the recommended range of maximum 7,400–8,000 cGy per each dosimeter. The dosimeter was irradiated with 200 cGy on a daily basis. The measurements were performed using a solid water phantom, as in Fig. 3. The readings were performed at the controlled room temperature of $22^{0}C\pm 1^{0}C$. Moreover, the readings were used to calculate the correction factor between room temperature and human body readings. The irradiation depth was 8.4 cm and a field size was 10.4×10.4cm.

#### B.3 Field size dependence

The influence of the field size on measurements outcome was tested using a field size dependence test. The DVS was irradiated with 200 cGy (different number of monitor units calculated in TPS) using the following field sizes: 4×4,8×8,10.4×10.4, and 16×16cm.

#### B.4 Postirradiation signal drift

According to the specifications of the manufacturer, the most accurate readings are obtained when the postirradiation measurement is performed 2–3 minutes after RT delivery has been completed. However, the question arises of what will happen if therapists take measurements before or after the recommended period of time due to unforeseen circumstances (e.g., helping patients, device or computer breakdown). Under such circumstances, the appropriate correction factors obtained from this experiment should be applied to obtain the actual dose. The test includes the irradiation of the dosimeter under standard conditions (defined as 200 cGy, 10.4×10.4 size at the reference recommended depth, 8.4 cm for this case), and postirradiation readings taken after 30 seconds, 1 minute, 2.5 minutes, 5 minutes, and 10 minutes.

#### B.5 Dependence on average dose rate

This test was designed to check constancy of the readings with various constant dose rates or accelerator output (i.e., dose per MU). Under these circumstances, the dosimeter reading should not vary. The experiments were performed under standard conditions using a solid water phantom. The dosimeter was tested with three average dose rates: 150, 300, and 600 cGy/min.

#### B.6 Linearity test

The dose linearity test included the irradiation of the dosimeter in the recommended range of the absorbed doses several times. The dosimeter was irradiated under standard conditions to deliver 150, 175, 200, 225, and 250 cGy, respectively. The dosimeter was tested only in the range of daily fractions for prostate IMRT treatments. For this test, the dose rate was constant.

#### B.7 Angular dependence (different gantry angle position)

**Figure 4:**
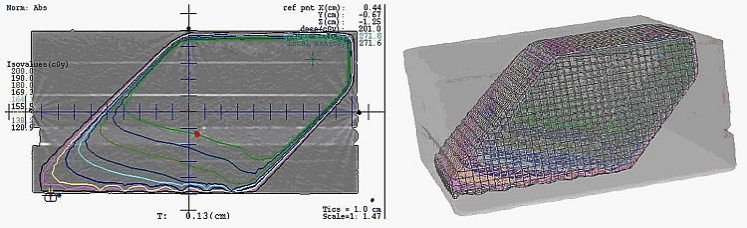
Dose calculation and distribution for the dosimeter inside the solid water phantom when a gantry angle was 45°; isodose line of 200 cGy cuts the dosimeter (red) in axial view.

The purpose of the angular dependence test was to characterize the DVS dosimeters' dependence on different gantry angles during the radiation exposure. The dosimeters were irradiated under standard conditions with different gantry angles: 0°, 45°, 75°, 90°, and 135°, where the beam with the 0° angle was an anterior–posterior field and the angle counting was in clockwise direction. The TPS was used to calculate the number of MUs to deliver the nominal 200 cGy to the measuring point. Consequently, the treatment depth varied from 5.3 to 9.9 cm for the gantry orientation of 45°, as in Fig. 4.

Using this methodology, we were able to evaluate not only the relative, but the absolute readings, with respect to gantry angle changes. The chosen gantry angles are close to the ones used in standard prostate IMRT treatments.

#### B.8 Angular dependence (different DVS angle position)

It was noticed that there was some rotational anisotropy for the dosimeters^(^ ^13^ ^)^ (i.e., rotation around the long axis). A multifield plan would possibly reduce this effect. However, if a single field is used, the anisotropy may be significant.

The purpose of the angular dependence with different DVS position test was to evaluate the DVS dosimeters' dependence on different DVS angles during radiation, as suggested.^(^ ^23^ ^)^ The dosimeters were irradiated under standard conditions with different DVS angles, where 0° angle was defined when DVS measuring point was towards the gantry. The angular orientation policy was the same as in the previous test. The dosimeter angles were: 0°, 45°, 90°, $-45^{0}$, $-90^{0}$, 135°, $-135^{0}$, and 180°. For this test, the treatment depth was constant at 6.3 cm. All plans were normalized to give 200 cGy at the measuring point of the dosimeter. Measurements for the constant treatment depth were used to correct the possible influence of the different treatment depths in the previous test.

#### B.9 Dose rate dependence

For this test, SSD was kept fixed, and we varied machine dose rate (MU/min). To test the dose rate dependence, the dosimeter was irradiated under standard conditions with dose rates from 50 to 500 cGy per minute in steps of 50 cGy. The real dose rate at the machine display was recorded, if the desired dose rate was different from the achieved one. The measurements were performed using a solid water phantom at room temperature. The readings were corrected for the temperature dependence factor.

#### B.10 Irradiation depth dependence

The dosimeters were calibrated to give accurate readings at depths greater than $d_{\max}$, within typical clinical depths ranging from 5 cm to 10 cm. Irradiation depth dependence tests confirm reading consistency within clinically relevant depths and assess readings which are outside recommendations. Due to patients' anatomy, there is a higher probability that the DVS can be placed at greater rather than shallower depths. We investigated the DVS readings for standard experimental setup, for 10 MV photon energy, and for the following depths: $d_{\max}$ and 5, 10, and 15 cm. The absolute dose at the specific depths was determined using TPS.

#### B.11 Effect of cone‐beam exposure to the dosimeter

The dosimeter is not calibrated for use in the kilovoltage (kV) range of photon energies. However, the effects of kV cone‐beam computed tomography (CBCT) imaging to the dosimeter were investigated. The readings were taken for a full 360° scan using the prostate‐specific CBCT protocol. Sometimes, when the patients have already been setup for the treatment, it is recommended to perform the pre‐RT DVS measurement prior to CBCT and then proceed directly to RT delivery. Consequently, to get accurate readings after the treatments, it is necessary to subtract the CBCT reading. This test was designed to evaluate DVS measurements associated with CBCT. The test was performed using three different dosimeters, and each dosimeter was exposed to kV radiation ten times.

#### B.12 A multiple reading effect

The dosimeters use a p‐channel MOSFET as the radiation sensing mechanism. The telemetric data acquisition system generates a low‐frequency magnetic field which can induce a fading of the measured signal. Due to that fact, the reading can be taken once per day. A multiple reading test was designed to assess the readings after ten consecutive irradiations under standard conditions, using a solid water phantom, and ten consecutive paired pre‐ and post‐RT data readings.

## III. RESULTS

The results of the tests described above are presented in this section. Whenever appropriate, correction factors are used to obtain accurate dose measurements. Based on the commissioning results, we adopted appropriate procedures for clinical implementation of the DVS system (described below).

### A. temperature dependence

**Figure 5:**
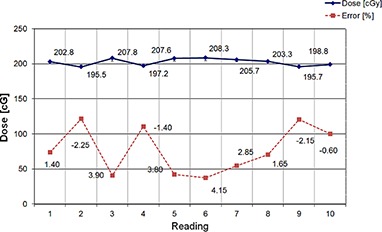
Dosimeter readings for test performed in water at the human body temperature for the nominal irradiation of 200 cGy.

The reproducibility test in water at human body temperature (37°C) showed a 1.4% absolute difference, with a standard deviation of 5.72 cGy (i.e., SD=2.9%). A mean range of measurements of 12.3 cGy (6.2%) was observed for each individual dosimeter. The skewness of the data was −0.2, which indicated asymmetry of data distribution. The negative skew indicates that the tail of the data on the left side of the probability density function is longer than the right side and a majority of the values, including the median of 202.8 cGy, lie to the right of the mean, which was 202.18 cGy. This reflects the factory calibration of the dosimeter. The dosimeter is always calibrated to overestimate the dose at the first part of the allowable dose limit of 8,000 cGy (from 0 to 4,000 cGy), and to underestimate the dose at the second part of the allowable dose range (4,000 to 8,000 cGy). We believe that the noted factory calibration method was necessary to ensure accuracy up to ±7% for the whole irradiation range. The major constraint affecting the calibration method was the significant fading effect in the MOSFET. Figure 5 shows the water measurements at 37°C.

The same test in water at 41°C showed slightly higher absolute values of the median (203.3 cGy; 1.6% higher) and the mean (202.36 cGy, 1.2% higher). Similarly, the skew was −0.3. The SD was 5.11 cGy, or 2.6%.

For both measurements, the kurtosis was negative (−2.8 and −1.6), which indicates a platykurtic distribution with a lower, wider peak around the mean (see measurements 5–8, Fig. 5), with a higher probability than a normally distributed variable of values near the mean. Bearing in mind the previous explanation about dosimeter calibration, this was expected.

When the dosimeters were irradiated at 37°C for the study, then their storage temperatures were maintained at 37°C. Frequent, rapid temperature changes can reduce the accuracy of the dosimeters. Our experimental setup was designed to exclude these errors.

Measurements at the controlled room temperature of $22^{0}C\pm 1^{0}C$ showed the median and the mean, 193.3 cGy (−3.3%) and 194.06 cGy (−3%), respectively; SD=2% (3.99 cGy). The test was performed using three dosimeters, which were irradiated for the whole useful range of radiation (0 to 8,000 cGy).

### B. Reproducibility

The reproducibility (or constancy) test shows that the average readings at room temperature were 3% lower than readings at human body temperature, with a SD=2%. This finding is in compliance with the specifications which note that readings at room temperature are 3%–5% lower, and with previous studies.^(^ ^14^ ^,^ ^17^ ^)^ The small positive skew of 0.3 indicates that the tail on the right side is longer than the left side, and the bulk of the values lie to the left of the mean. This difference, compared to the previous test, is expected because the dosimeter was irradiated to 8,000 cGy. This information means that the falloff of the readings is more rapid after the dosimeter has received a radiation dose over 6,000 cGy. A lower negative kurtosis (−0.5) was observed which means that above 8,000 cGy, the factory calibration eliminates wider peaks around the mean.

**Figure 6:**
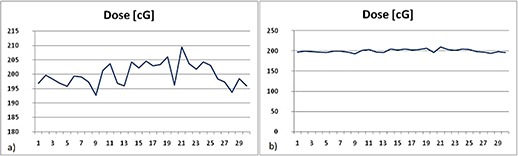
The reproducibility test: (a) the individual readings have relatively wide range; (b) absolute values are still within specifications

Figure 6(a) shows a wide range of the readings, 16.8 cGy (8.4%), whereas Fig. 6(b) shows that, on the average, the readings are within the vendors' specifications. This result will direct the implementation procedure. According to the results, it is not useful to analyze and to make the action point based on only one reading, but on several average readings.

A rapid falloff of the readings was noticed when the dosimeter received an additional 4,000 cGy after the initial 8,000 cGy. The readings decreased by about 0.28 cGy per every 100 cGy of radiation. It means that the nominal readings dropped by 11.2 cGy, while the dose of radiation increased from 8,000 to 12,000 cGy.

### C. Field size dependence

Field size dependence was not observed. After delivering 200 cGy to the DVS, using field sizes of 4×4,8×8,10.4×10.4, and 16×16cm, it was noticed that the mean was in the range of 199.29 cGy to 200.44 cGy, with SD less than 1% for all measurements. Consequently, it can be concluded that field size does not influence the readings for the predefined field sizes. This fact was expected, because the measuring point of the dosimeter was small.

### D. Postirradiation signal drift

**Figure 7:**
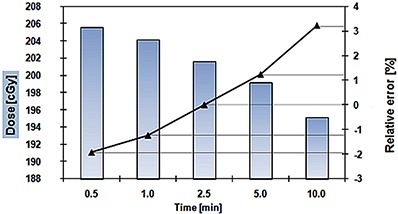
Postirradiation readings depend on time when the readings were taken. The triangular symbols denote the relative errors.

The results of postirradiation signal drift are presented in Fig. 7. The vendor's recommendation was to perform the postirradiation readings 2 to 3 min after the fraction had been delivered. However, it is not always possible to do so. The columns show the absolute readings. It was confirmed that the readings taken after 2.5 minutes were the closest to the delivered 200 cGy, with absolute error within 0.42%. It can be noticed that the readings recorded earlier than 2.5 minutes following RT are overestimated, and the measurements taken later than 2.5 minutes after RT are underestimated.

The black curve shows the relative error during time, normalized to the readings taken 2.5 min after irradiation. If the postirradiation readings were taken before or after the predefined time, correction factors must be applied in order to assess the delivered dose more accurately. The corrections are −2.8%, −2%, 0.5%, and 2.5% for the readings taken after 0.5, 1, 5, or 10 min, respectively. The reported results show a time dependence higher than previously measured.^(^ ^14^ ^,^ ^17^ ^)^

### E. Dependence on average dose rate

The experiments show that the DVS readings do not vary significantly with the average dose rate. The ratio of maximum to minimum readings was within 1.3% of delivered 200 cGy for each predefined dose rate (150, 300, and 600 cGy/minute). However, it was noticed when the dose rate increased, the dosimeter reading increased as well; for instance, for dose rate of 150 cGy/min the average reading was 198.6 cGy, and for dose rate of 600 cGy/min the average reading was 201.6 cGy. Consequently, the measured integrated signal tends to increase with the dose rate. The SD of the measurements was 4.98 cGy (i.e., 2.5%).

### F. Linearity test

**Figure 8:**
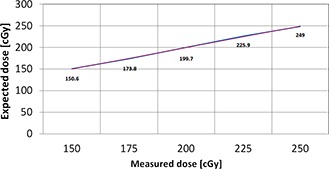
Linearity test for the implantable dosimeter: blue=line connect measured values; red=trend line.

The linearity test was performed for a range of calibrated doses from 150 to 250 cGy. The readings showed good compliance with the calculated dose for each point, with standard error ±1.1% and SD <2% (Fig. 8). The measurements at controlled room temperature confirmed that the correction factor of 3% should be used to correctly obtain absolute readings.

### G. Angular dependence (different gantry angle position)

The angular dependence test was designed to discover possible inconsistency with the delivered dose and dosimeter readings during standard 3D or IMRT isocentric treatments in which the gantry rotated around the patient. In this case, the isocenter was always at the dosimeter measuring point, as in the SAD treatment technique (1.3 mm from the dosimeter top end). The median and the mean for all measurements were 200.74 cGy (+0.4%) and 200.63 cGy (+0.3%), respectively. The SD was 2.88 cGy (1.4%). The skewness was 0.59, which indicates that the tail of the right side of the data distribution was longer than the left side and the bulk of the readings lie to the left of the mean. This was because the specific dosimeter was already irradiated with more than 4,000 cGy at that moment. The data were in a platykurtic distribution which indicated a uniform data distribution (the kurtosis was −0.24).

Moreover, the mean for the readings taken at gantry angles of 0°, 45°, 75°, 90°, and 135° were 200.94, 200.39, 198.98, 199.2, and 199.24 cGy, respectively. The maximum error was less than 1%, with a maximum SD=3.61cGy (1.8%) for the gantry angle of 45°. It can be concluded that different gantry angles did not influence the DVS readings.

### H. Angular dependence (different DVS angle position)

**Figure 9:**
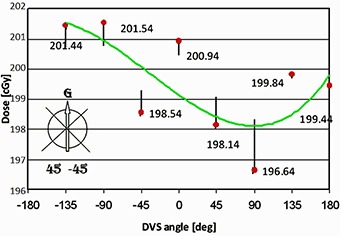
Readings (red) and absolute errors (vertical lines) for different dosimeter positions. Green line represents the polynomial trend line for the readings; G denotes gantry; arrow represents orientation of the dosimeter.

The average readings with absolute errors for different dosimeter positions are shown in Fig. 9. In the lower left corner of the figure, the gantry and dosimeter orientation are displayed. The green line is a third‐order polynomial trend line. It can be seen that the readings are dependent on the dosimeter orientation. As expected, the most accurate reading was obtained when the dosimeter angle was zero. The readings decreased for the angle range from 0° to 90°, and then increased again when the dosimeter approached 180°. Similarly, when the dosimeter angle was in the range of 0° to 135°, the readings first decreased and then increased.

However, the maximum error was −1.68% for the dosimeter angle of 90°. The median and the mean for all readings were 199.64 and 199.57 cGy (−0.2% and −0.2%), respectively. The SD was 1.73 cGy (0.9%). The reported results are similar to ones in Briere et al.^(^ ^14^ ^)^ for rotation around the long axis of the dosimeter.

### I. Dose rate dependence

Significant dependence on the dose rate was not noticed, as evidenced there was no correlation between dose rate and reading. The results are given in Table 1. The median and the mean were 199.29 and 198.66 cGy (−0.4% and −0.7%) for the measurements. However, a higher dissipation of the results was recorded: SD=3.75cGy (1.9%) and range of 13.85 cGy. More measurements correspond to a larger range, but the average readings were close to the previously obtained value. This fact influences the implementation methodology for clinical use (i.e., the weekly average readings were analyzed for clinical decision making, such as replanning, instead of the daily fluctuation of the readings).

**Table 1:** Dose rate dependence results.

	*DVS2 1005377*	*200 cGy*	*233 MU*	d=8.4 cm
	*FS:* 10.4×x10.4			
*Energy*	*Planned Dose Rate [cGy/min]*	*Real Dose Rate [cGy/min]*	*Value [cGy]*	*% Diff.*
6 MV	50	38	200.34	0.17
	100	75	196.94	−1.53
	150	150	191.94	−4.03
	200	150	195.34	−2.33
	250	305	200.64	0.32
	300	306	195.64	−2.18
	350	305	199.74	−0.13
	400	305	198.84	−0.58
	450	613	202.74	1.37
	500	613	204.44	2.22
		Median	199.29	−0.4
		Mean	198.66	−0.7
		St.Dev.	3.75	1.9

### J. Irradiation depth dependence

This test revealed that the readings decreased with increasing depth, about 0.3%/cm from $d_{\max}$ to 15 cm depth. The readings at $d_{\max}$ were 3.25% above the delivered dose, so the readings at a depth up to 10–12 cm were still in the range of 1% error. During the analysis of ten randomly chosen patients, it was observed that the separations to the prostate center or to the dosimeters were from 9.4 to 15.3 cm for the anterior side, and from 8.4 to 15.9 cm for the posterior side. The findings in this experiment deserve further attention, and an independent study of this topic will be part of future work.

### K. Effect of CBCT exposure to the dosimeter

**Figure 10:**
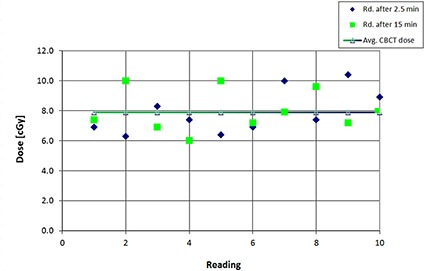
Readings after the dosimeter was exposed to CBCT: (a) blue – postirradiation readings were taken after 2.5 min; (b) green – postirradiation readings were taken after 15 min. Y‐axes represent the change in the device signal with radiation.

The average dose reading was 7.89 cGy (SD=1.46 cGy) due to CBCT imaging using the pelvis protocol. The postirradiation measurement was taken at 2.5 minutes. Sometimes, it is necessary to take the postirradiation reading after the IMRT treatment, which is approximately 15 minutes after the CBCT. In that case, the average dose reading was similar, 7.40 cGy (SD=1.48cGy). This means that the readings taken after the kV irradiation were affected by a postirradiation signal drift in a similar manner as in the case of irradiation in the MV range (Fig. 7). Figure 10 represents the results of 20 readings using CBCT; half of them were taken 2.5 minutes after irradiation (average value of 7.89 cGy) and the other half at 15 minutes postirradiation (average value of 7.40 cGy). Since the device has not been calibrated for diagnostic energies, the average dose reading of 7.89 cGy is not an accurate dose value. However, the average number plays an important role in measuring accurate patent dose, since sometimes predose readings have to be taken before CBCT scanning. Consequently, to get the accurate readings, the CBCT radiation‐induced signal needs to be subtracted from the postpatient irradiation‐induced signal to obtain the correct dose from the therapy procedure.

### L. Multiple reading effect

**Figure 11:**
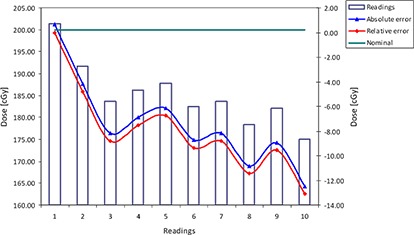
Rapid falloff noticed after ten consecutive measurements.

The readings after ten consecutive irradiations were represented in Fig. 11. A rapid falloff was noticed instantly at the second consecutive reading. Based on the results from this test, it was confirmed that the implantable dosimeter could not be used to measure the dose after each beam in a clinical setup, or several times during the day for research purposes.

### M. Clinical implementation

The DVS program was implemented for patients undergoing prostate cancer RT at our institution. The dosimeters were placed in patients in the dorsal lithotomy position, using transrectal ultrasound guidance. Transperineal placement of fiducial markers into the prostate, using ultrasound guidance, was shown to be safe and to have low incidence of infections.^(^ ^24^ ^)^ The dosimeters were identified on the helical planning CT and the MOSFETs were identified as points of interest in the TPS, as described previously.^(^ ^15^ ^,^ ^25^ ^)^ The doses were calculated in the TPS for each dosimeter in the final IMRT plan. The calculated doses were used as the reference dose to compare the daily measured doses.

**Figure 12:**
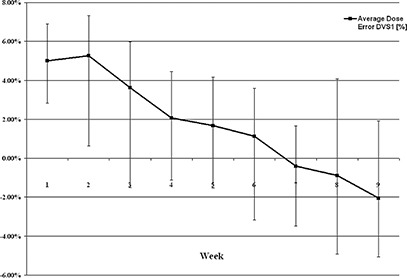
Average deviation from expected dose for 17 IMRT prostate patients during the RT course.

A threshold of ±7% was used to identify clinically significant dose discrepancies. Two or more discrepancies ≥7% within any given week, or any discrepancies ≥10%, prompted an investigation by the radiation oncologist and medical physicists. The investigation included careful review of all CBCT images, including position and angle of the dosimeters and any changes in pelvic anatomy, adjustment of imaging dose as recorded by the therapists, and review of the treatment plan in the TPS. When a potential cause of dose discrepancies was identified, appropriate steps were taken by the treatment team, which sometimes involved replanning the case.^(^ ^26^ ^)^ Detailed analysis of the patient dose measurements under IMRT beams can be found in Den et al.^(^ ^27^ ^)^ Figure 12 shows percentage deviation of prescribed dose for the 17 IMRT prostate patients during the nine weeks of treatment.

## IV. DISCUSSION

Herein we described the clinical commissioning and implementation of the DVS with permanently implanted *in vivo* dosimeters.

The previous results show that the average measured dose is quite close to the true expected dose of 200 cGy, with the SD falling between 1.4 and 3.6%,^(^ ^14^ ^)^ which is in agreement with SD from 2% to 3% obtained in this study. The Briere study^(^ ^14^ ^)^ found that the measured dose tends to be slightly underestimated for smaller doses, while it tends to be slightly overestimated for larger doses. Similar results were recorded in our clinical studies (see Fig. 12 and Results Section A), where asymmetry of data distribution was noticed. It was reported by Scarantino et al.^(^ ^15^ ^)^ that the error frequency at >8% was as high as 47%, 29%, and 21% for lung, prostate, and rectal tumors, respectively. Phantom studies presented here did not confirm this fact, but additional clinical studies^(^ ^26^ ^,^ ^27^ ^)^ showed higher discrepancies between measured and expected doses in clinical settings. Studies by Black et al.^(^ ^16^ ^)^ and Beddar et al.^(^ ^17^ ^)^ confirmed that bounding cumulative errors due to setup, planning, and machine output within a ±5% level are achievable, but deviations from the targeted dose often exceeded ±5% error in patients, as demonstrated in Beyer et al.^(^ ^18^ ^)^ Beddar et al.^(^ ^17^ ^)^ found that reproducibility within 2% could be achieved in a well‐controlled experimental environment, which is comparable with 1.4% absolute difference obtained in this study (see Results Section A). The recorded temperature dependence^(^ ^19^ ^)^ was within 2.0% (25°C–37°C) vs. 3% in this study. A strong angular dependence^(^ ^19^ ^)^ was observed for gantry incidences exceeding ±30°. It was revealed in this study that the maximum error was −1.68% for the dosimeter angle of 90°. The implantable dosimeters are calibrated to be irradiated with 150 to 250 cGy fractions once per day. More frequent irradiations give suboptimal results, as concluded in the Results Section B above.

Measurements performed for commissioning show that the dosimeter, if used within specifications, provides sufficient accuracy for its intended use in clinical procedures. The postradiation signal drift, temperature dependence, variation of reproducibility, and rotational isotropy could be encountered if the dosimeter is used outside the manufacturer's specifications. Clinical implementation excluded the influence of these variations (for instance, postreadings were taken after 2.5 min, when variation was less than 1%). Furthermore, to obtain the correct absolute dose during the studies at room temperature, a correction of 3% should be applied. Due to the fact that the falloff of the reading increases when the received dose is higher, it is suggested that phantom studies should be performed with dosimeters which received doses less than 6000 cGy. In addition, correction of 0.3%/cm can be used to correct the decrease in readings from $d_{\max}$ to 15 cm depth, and if the clinical setting requires CBCT prior to treatment, the average of 7.40 cGy should be deducted from the final measurements.

The DVS readings, when properly analyzed, can alert the physician when a different dose is being delivered, which enables the physician to determine whether the source of the problem is due to patient or organ motion, organ deformation, errant calculation, or machine error. The system is currently implemented with a weekly tolerance of ±7%.

## V. CONCLUSIONS

The dosimeter can be used as a tool for quantifying dose at depth, as well as to evaluate adherence between doses from the treatment planning and the delivered dose. Dependence of small filed sizes (less than 4×4cm) would be of future interest, especially when small radiation segments are used during IMRT. The DVS dosimeter does not identify the specific cause of the difference in dose, but it can reliably alert the physician that a deviation between the planned and delivered dose has occurred. As such, the dosimeter can act as a fail‐safe device with the potential to catch an over‐ or underdose situation before the mistake could be repeated.

There are important advantages to DVS dose verification. It can be reliably used for the verification of any possible change of the dose to the target or nearby organs. The system is capable of keeping a permanent patient dose record by monitoring the daily dose delivered. Based on the dosimeter readings and trends, the dose changes might be predicted. If the weekly average readings were outside the clinically implemented tolerance, the physician can investigate the patient position, clinical protocols, internal anatomy, and treatment plan, then apply corrections, if necessary. Furthermore, the DVS system provides medical physicists with an independent QA verification of machine performances. The extensive commissioning and implementation strategy detailed above can improve the usage of implantable dosimeters and may lead to improvements in patient treatment outcomes.
